# Technique of Conservative Pulp-Treatment

**Published:** 1894-11

**Authors:** Louis Jack

**Affiliations:** Philadelphia


### International Dental Journal.

Vol. XV. November, 1894. No. 11.

### Original Communications.[1]

### TECHNIQUE OF CONSERVATIVE PULP-TREATMENT.[2]

BY LOUIS JACK, D.D.S., PHILADELPHIA.

Gentlemen,—My lecture before you this evening is upon a subject which, to students, must be of considerable interest. It is the more agreeable to consider this operation before you for the reason that it gives the opportunity to deal with the most minute details of treatment. It is presumed that the least minutiæ in the management of the exposed pulp, and of the considerations connected with the treatment thereof, will not fail of receiving your attention. Whatever previous instruction you may have had in this field, you must welcome varying light upon every feature of the treatment.

The preservation of the vitality of the exposed dental pulp has from an early period in the history of dentistry been a desideratum,—first, as a means of securing the avoidance of pain, and, secondly, because the devitalized tooth becomes of less value, for the reasons that its appearance is impaired, its cohesion is gradually lessened until at length it is lost by fracture, and in many instances

[1] The editor and publishers are not responsible for the views of authors of papers published in this department, nor for any claim to novelty, or otherwise, that may be made by them. No papers will be received for this department that have appeared in any other journal published in the country.

[2] A lecture delivered before the class at the Department of Dentistry, University of Pennsylvania.

the abnormal condition arouses disturbance of the surrounding parts.

The views held upon the probability of success in the conservative treatment of the pulp, it must be admitted, are at the present time in a very discordant state. As you may have observed, some claim a very large percentage of successful results, and others denounce any attempt at such treatment. How are we to reconcile these counter views, held in each case by intelligent and conscientious men. The causes of this disparity of opinion will appear at least by inference as the consideration of the general subject advances; only this may be stated here, that either the treatment has not been conducted with the required carefulness or the attempts have been made upon cases where this treatment was not applicable. The differences in the conditions of various exposed pulps are as great as that between simple and compound fractures of the bones, in the latter of which the prognosis is in general doubtful; and I shall attempt to define the distinction between salvable pulps and those doomed to inevitable loss, and thus to present to you some lines upon which to establish the prognosis of any given class of cases.

At the very outset let me state to you that, while the treatment of the pulp in this manner is not difficult, it is one of the most delicate operations in dentistry, requiring the truest hand, the lightest touch, the greatest patience, and the utmost consideration for the sensibility of the patient. Furthermore, it necessitates a careful observation of all the symptoms and presented conditions, with a rational analysis of these. He who has the physical and the mental qualifications need not fear to enter this field if he will keep within the limitations governing success.

There are several general propositions in reference to the pulp necessary to be kept in view as guiding principles. These are:

(*a*) The pulp of the tooth in the earlier condition of simple encroachment by caries is not a very sensitive organ, as it permits the approach of the carious action without in the majority of instances indicating by the presence of pain that it has been invaded, and it tolerates in this condition light instrumental contact with but little indication of pain.

(*b*) It is more impatient of compression than of any other form of irritation.

(*c*) It is intolerant of drugs, and accepts only the milder kinds of medication, and of such character as to be soluble in water.

(*d*) In the earlier stages of exposure the elements of the organ involved are its nervous filaments, which are hyperæsthetic from the hyperæmic state of the organ adjacent to the point of exposure.

(*e*) That at this stage the pulp becomes impatient of cold, and may indicate the encroachment of caries by subjective (reflected) pain in other branches of the trigeminus.

(*f*) That later on, unless these conditions are subdued by treatment, congestion of the organ takes place, when objective disturbances arise, which indicate that the point of danger has approached. Soon thereafter hopes of successful treatment may vanish.

In a well-regulated practice, where the patients are under frequent observation and have regular and periodic care taken of the teeth, the pulp exposure which takes place should be of the simpler kind, and should be placed under treatment so early after the carious action has affected the pulp that the prospects of successful treatment are not remote.

But when neglected cases appear, the history of which has been obscure, and when the patient is driven for relief by the occurrence of objective symptoms, the prospects of success are of doubtful character. The contrast of these two classes will throw some light upon the frequent absolute failures of attempts to treat the exposed pulp, and accounts for the discredit which has been cast upon conservative treatment.

At the commencement of the treatment to restore the lost tissue in any given carious tooth, except in the obviously very small cavities, the probability of encroachment upon the pulp should be a supposition, and each step should be made with reference to this probability. The destruction of the dentine is frequently surprisingly deep, or the cornua of the pulp often is acutely pointed and may be unexpectedly encountered. Therefore, in what may seem simple cases, cautious approach should be made towards the bottom of the cavity.

The opening of the cavity should be effected by instruments which will not easily enter the cavity, and the softer caries removed in a manner which will not induce pressure of the decayed matter upon the pulp, as once this organ is trampled upon, no after-degree of care will avail to subdue the irritation which is liable to arise. The subsequent irritation caused by active pressure in this manner probably is due to disturbance of the pulp-tissue in its relations, caused by its laceration from the walls of its cell; which may be the consequence of the feeble connection between the pulp and the walls of the chamber.

For the same reason, in the removal of the caries the excavation should be first carried on at the sides of the cavity, and also along the margin of the cervical wall in proximal cases. Then the carious matter nearest the pulp should be carefully peeled off without pressure and without irritation. In this manner a pulp may be uncovered and the cavity cleansed of carious matter without contact being made with the pulp. To do this is the acme of skilful preparation. To uncover the dental pulp without pressure and without making contact with it is more than half the battle, if so peaceful a proceeding can be so designated.

The instruments for removing the caries should be of thin edge, very sharp, and always having cutting surfaces which are rounded, since angular or square-ended excavators are liable to make exposures unnecessarily. It is important that the direction of movement of the excavators should be from the cervix towards the coronal part,—in other words, by drawing cuts instead of pushing cuts. The difference in the excitement of pain between these two methods of cutting is surprising, and can only be appreciated by those who have experienced the comparison upon their own teeth. The probable reason for this is that the force of the pushing cut is necessarily greater. It certainly causes more pain at the moment, and the cleansing in this manner is followed by greater after-irritation. Patients will complain at the time of reflected pain being caused by pushing cuts, and may feel the effects in the trifacial ganglion.

It must be plain to you all that any mode of procedure which increases the local irritation in the preliminary procedure of a pulp treatment must be deleterious to the outcome of the case. Therefore I emphasize the proper direction of the cuts. I would also state that the danger of making unnecessary exposures and of forcing the instruments upon the pulp are greater under push cutting. You will not fail to consider that the insertion of burring instruments in this class of cases would be entirely unadvisable, since the infliction of some compression would be unavoidable.

Now, an interesting question appears. A cavity may be sufficiently deep to cause an exposure; it is carefully cleansed, and the cornua of the pulp is not observed. There may have been evidences of irritation. How shall we determine whether there is a real but minute exposure, or whether there is a safe amount of tissue to protect the pulp beneath the stopping-material?

I would state here, somewhat out of place, that I consider it better in all cases to remove all soft caries, feeling it the safest side on which to choose, than to introduce a filling over the ultimate layer of decay with the hope that after all the pulp may not be actually reached and that the last layer of decay may serve, when disinfected, as a better non-conductor than some foreign substance. This opposite view has caused me grief on many occasions, and will cause you to fall into the same pit if you will allow yourselves to be deluded by it. Therefore, I advise you to remove the soft caries, stopping the excavation when the tissue begins to have a reasonable degree of consistence. Only experience will give you the facility to determine the safe line.

Going back again, how shall we be sure the pulp is not exposed, in case it does not appear to the vision to be so? One method is to cross-hatch the cavity by a very fine explorer; this is done by holding the instrument very lightly and skimming it over the surface in close parallel lines in two directions. If exposed, the instrument at the point of encroachment will lose its resistance or will drag the point of the cornua, as the case may be.

Again, while there may be no visual evidence of exposure, the certainty of it may be shown during the preparation or the testing by a peculiar expression of the face of the patient, different from that manifested by the cutting of the most exquisitely sensitive dentine. This change of the countenance, accompanied with a slight start of the features, may occur without the recognition of pain. I am unable to give you language to describe it better. This indication sometimes appears before nearly all the caries is removed, and is probably caused by some dragging of the apex of the cornua in the disturbance of the carious dentine.

There is still another test of exposure which may be applied in doubtful cases, and which may often be used to determine the probability of exposure before the treatment has commenced. This is of assistance when the cause of reflected pain is occult, and where we have to determine whether the pain, amounting almost to a tic, is caused by a disturbed pulp, or is brought on by malarial influence or a visitation of gouty neuralgia.

I have already called your attention to the influence of cold upon the tooth in a hyperæsthetic state of the pulp. The test by cold is of the greatest importance in the diagnosis. The impatience of the tooth to cold in the earlier disorder of the pulp is an aggravation of what Dr. Black has called the temperature sense of the teeth. This sense of the teeth to cold I may state to you is very various in different individuals; some being able to drink the coldest water and to craunch ice with impunity, while others whose teeth are sound are impatient of the application of ordinarily cold water if applied directly to the teeth.

This irritability of the teeth, whether it appears naturally or in an aggravated form, is conveyed through the enamel, and in the latter case is a positive sign of disturbance not to be mistaken. By means of it the earliest stages of pulp-disturbance may be determined by isolating the suspected tooth by passing it through a piece of rubber. If carious, the cavity should be temporarily closed with varnished cotton when cold water is applied to the enamel. In making this test the adjacent sound teeth should be tested to attain a comparative result. This is necessary, because of the varying degree of normal sensibility of different persons. The use of this is of value to determine whether any given irritation in doubtful cases is dependent upon the condition of the teeth. If the case is one of malarial or gouty origin, the teeth do not abnormally respond to the cold test. Another diagnostic sign is the occurrence of the pain in the evening. Whereas, neuralgic attacks are more frequent, when dependent upon malaria or gout, in the early hours of the day. We come now to

### THE IMMEDIATE TREATMENT OF THE UNCOVERED PULP.

When accidental exposure has been made, tincture of calendula, one part to four of water, should be applied as soon as bleeding has ceased. The pulp should then be immediately dressed and capped in the manner to be described later. If only a slight injury has been inflicted, the cavity may be filled at once with metal, having regard to the strength, the placement, and the fixation of the cap used to defend from compression, as will later be described. Here the fixation of the cap is best made by covering it with a broad mat of gold-foil; after adapting this to the margins one may proceed to complete the filling. In respect to this class of exposures there should be no concern as to the success of the treatment, providing the pulp was in a healthy condition at the time of the accident.

When the pulp has been fully uncovered, as previously described, the cavity should be washed clean with tepid water, securely protected from the fluids of the mouth with rubber dam, dried, and lightly filled with a pledget of lint saturated with a mild disinfectant. From your knowledge of the causes of caries and of the concomitant invasion of the zone of dentine immediately beneath the caries by bacteria and micrococci you will recognize that some means of sterilization must be resorted to. It is necessary in the treatment of ordinary cavities, hence you will readily perceive how much more it is here required. On account of the impatience of the pulp to medication, we have to be very careful in the selection of the sterilizing agent. There is none with which I am acquainted that is less irritating than hydronaphthol. This should not be stronger than 1 to 300 parts of water. The saturated pledget of cotton may remain in the cavity during the procedures of the preparation of the dressing paste, the selection of the cap, etc.

When these preparations are complete the cavity should be again dried, the drying being finished by a few puffs of warmed air. The point of exposure and the adjacent dentine is now touched with a tent of lint, filled with carbolic acid and oil of cloves, āā. The effect of this is to coagulate to a superficial degree the point of exposure. This practice is largely empirical. It may be avoided in cases where no disturbance has previously existed; but where there are evidences of irritation, it has appeared by my experience to be indispensable.

The first impression made upon the minds of those who have been taught that carbolic acid is an irritant to pulp-tissue is that this proposition would appear to be questionable. On this point I have had much experimental knowledge. In the treatment of earlier cases under the prejudice that carbolic acid was irritating, I employed a weak solution of this agent. The results were not favorable, for the reason that in the dilute form it did not coagulate the surface of the exposed point, and was therefore liable to be absorbed, becoming consequently irritating to the pulp.

Pure, or nearly pure, carbolic acid combined with oil of cloves, as here recommended to you, at once produces slight coagulation of the surface of the organ and appears from the absence of irritation to enter no further into the tissue. You will not fail to observe the sterilizing and anæsthetic value of this combination.

In respect of the sterilization of the dentine, questions may arise in your minds whether this may be sufficient to cover the necessities of the case in this respect. First, whether complete sterilization is thus effected; second, whether the pulp may not already have become so far inoculated by bacteria as to render its normal condition an impossibility; and whether, under these circumstances, attempts of superficial sterilization of the dentine can be of any service.

You will not fail to keep in view that the treatment is confined to cases in which it is evident the pulp-tissue is probably not under much irritation. It is hyperæmic, and consequently in a hyper-

æsthetic state congestion has not occurred, nor does inflammation exist. Therefore, the inference is reasonably certain that after the soft caries is removed we may safely sterilize the surface of the dentine and leave the vital force of the pulp to take care of whatever slight bacterial invasion may have reached that tissue. I assume here that your instruction has included the observed fact that healthy tissues have the power of mastering the invasion of non-pathogenic bacteria.

### THE CAP.

A prominent feature in conservative treatment of the pulp is the means to protect it from pressure, and because of this necessity the means mostly used have given this mode of treatment the appellation of "capping the pulp." The object of the cap is the avoidance of compression. Various means have been used to accomplish this. A common method has been to cover by a dressing the point of exposure; the bottom of the cavity being then lined with oxychloride of zinc or phosphate of zinc, a coating of gutta-percha varnish being interposed between the dressing and the protective material. As this means is complicated, somewhat uncertain, not applicable in shallow cases, and in those very difficult of access, I have preferred to depend upon a metal cap as being simpler and better under control than the above described method.

This kind of cap not only insures to prevent compression, but serves to contain the dressing, which completely occludes the space between the cap and the pulp. These caps may be made of thin platinum or of gold, the former being preferable. In form they should be circular to meet the generality of cases, and oval to accommodate broad exposures. They are most easily made by punching them out with the round and oval leather punches of the hardware shops upon the end of a block of hard wood. This action gives them sufficient concavity for the purpose. For ordinary purposes they should be quite thin, but when it is considered safe to use gold fillings the cap should be sufficiently thick to resist the force of the packing.

### THE DRESSING.

It is necessary that the space under the cap should be completely closed, otherwise it would fill with fluid effused from the pulp, which quickly would undergo putrefactive changes resulting in the evolution of gas and consequently of compression of the organ beneath. In the earliest attempts at capping the pulp this became the condition, the only thought then being to protect from the pressure of the instruments. It was then generally supposed that the pulp underwent projection into the space and became strangulated, but all the indications now point to compression by gases being the cause of the almost immediate disturbance.

The dressing I have found most acceptable is formed by rubbing up oxide of zinc into a paste with carbolic acid and oil of cloves of equal measure. The consistency of this should be such that, while being so plastic that the paste will flow out around the cap when it is gently pressed in position, it will not be so thin as to flow out of the cap when it is held on its edge.

The ingrediency of the dressing is based upon these considerations. The menstruum is antiseptic, and possesses some anæsthetic value. It also remains unchanged within the space, and in time becomes, from the dissipation of the menstruum, somewhat firm in its character. The therapeutic action of the menstruum is mild, and is employed for the reason that it is slowly given up by the oxide of zinc, and, therefore, makes an acceptable dressing.

I would call your attention to the value of the combination of hypophosphite of lime with zinc oxide, āā, with the previously stated menstruum. This I have used in many of my earlier experiments, and have lately returned to it in cases of a less promising character than those considered within the lines of easy treatment.

### PLACING THE CAP IN POSITION.

Placing the cap in position is a step in the treatment requiring care. It should be assured that it is of sufficient size to pass well beyond the borders of the exposed organ, and in the proximate cavities it should cover the pulp-wall of the cavity without intruding upon the marginal walls. If there is a single exposure it should be round; if two coronæ are exposed, either two caps should be laid or one oval one employed, as may best suit the case. In molars, usually where two points are exposed, two caps are generally best; in the bicuspid one oval one under the same circumstances. The cap should be inserted edgewise in such manner that as it is laid in place the excess of dressing may flow out at the margin towards the operator. This is to prevent undue pressure, and to prevent air being included beneath the dressing, which would prevent complete apposition of the dressing with the pulp.

In cases of easy access the cap may be laid in place with fine-pointed pliers,—notably the Bogue plier; but, in the majority of instances it is preferable to previously coat the convex side of the metal with yellow wax, when, with an instrument adapted to the case, it may be carried into position, and then placed in the manner described. It should next be pressed into position with sufficient force to bring the margins in contact with the dentine. Any excess of dressing should be taken away by light touches of an excavator, and when the cavity is to be filled temporarily it is better to fix the cap in place by flowing over it a little chloro-percha, which, when dried, prevents disturbance of its position in the filling procedure.

You should be careful that when the pulp is found exposed in a depression, as occurs sometimes in the molars, this depression should be filled nearly or quite to a level with the floor of the cavity by taking a little of the dressing upon a suitable instrument and carefully filling this point; otherwise, when the cap is placed, the paste may not find its way into contact with the pulp. In a few instances, in my earlier experience, I have been obliged to go over the case and recap on account of this difficulty.

At the moment of placing the cap, as the paste is yielding under the gentle pressure of forcing the edges of the cap into contact with the dentine, a little pain will be observed; but, unless the paste is too stiff, no compression of the pulp will be caused.

### FILLING THE CAVITY.

Whether the cavity shall be filled temporarily or permanently depends upon the prognosis. This, as you will perceive, depends upon the constitutional conditions and the state of the pulp at the time of treatment.

To those of small experience in this line of treatment it would not be safe to attempt permanently stopping the cavity, except in accidental exposures, and in cases where no previous disturbance can be elicited. Even in the latter class it is generally best to delay permanent closure by a conductor of heat, until after an experience of a year or more with a non-conducting stopping. At the end of this time the filling may be nearly all removed, care being taken not to disturb the capping, when, with suitable precaution, a metallic filling may be inserted.

I have many capped pulps belonging to class *a* beneath gold fillings, where they have remained, without the occurrence of any irritation, for many years. But I must remind you that the utmost caution must be used in making the selection of cases to be filled permanently at the time of the capping of the pulp.

In the majority of instances it is safest to fill the cervical part with gutta-percha stopping, carrying the material over the cap, and then to complete the filling with zinc phosphate. In this way, with an occasional renewal of this temporary work, cases may be carried forward from ten to fifteen years.

They may, however, be closed permanently and safely after an experimental trial of five years where no irritation has appeared.

This brings us to consider the conditions which take place with the pulp after the procedures which I have described before you. Concerning this there is no certain data.

Obviously the most desirable result would be the conversion of the exposed surface of the pulp into secondary dentine. This result I have known to take place within the period of two years, and I have opened for examination cases which have been capped, without the occurrence of any disturbance beyond the occasional impressibility by cold for periods as long as fifteen years, where no such change had taken place.

That in many cases recovery is by secondary deposits my records present a considerable number of instances. It is not remarkable, however, that pulps may remain in a state of quiescence for years, when it is considered that in slowly-advancing caries the pulp will often be exposed for long periods without the occurrence of any signs of irritation, unless, by the position of the mouth of the cavity, the pulp has been subjected to the pressure of food.

It may be concluded that, whether the pulp becomes protected by secondary deposits or acquires complete quiescence, conservative treatment in these cases has considerable advantages over immediate devitalization. Still, in this connection, I must repeat the necessity for the careful selection of subjects to be treated in this manner, and also for proper regard to the apparent condition of the pulp itself. To aid you in this discrimination I give you a classification of conditions.

### CLASSIFICATION OF CASES OF EXPOSURE.

(*a*) Where no previous observable disturbances can be elicited.

(*b*) Where the tooth has been impressed only by the application of low temperature.

(*c*) Where, in addition, reflected pain in related parts has been observed.

(*d*) Where the tooth has become subjected to impressions by heat.

(*e*) Where continued objective disturbances appear, such as soreness to touch, or local pain of spontaneous character or pulsations.

As the result of long experience, I am forced to consider classes *a*, *b*, and *c* entirely admissible to treatment, and also, problematically, class *d*, if taken early.

Class *d* when advanced, and class *e* under any conditions and circumstances, whether presented in the states mentioned or having become so, are fit subjects for devitalization; any attempts to conserve these usually resulting in lamentable failures, suffering to the patient, and impaired confidence in the judgment of the operator.

It is important here to consider the influence of the physical endowments of the patient upon the conservative treatment of the pulp. For some persons the treatment is followed by the happiest results; no impatience of the operation appearing, and even cases somewhat unpromising doing well. Again, with others, every case however simple goes down the gamut to class *e* in spite of every care.

The first constitutional condition favorable to success, as you will at once agree, is that of soundness. As to what are called temperamental indications when the subject is of good health, I should exclude only the lymphatic, and more particularly the bilio-lymphatic. These latter do not respond to pulp-treatment in any conditions which occur to them; and in reference to their exposed pulps the probabilities are, that in the sluggish condition of the parts involved the organ is early invaded by bacteria, and such changes have quickly taken place in the anatomical elements of the pulp as to render all hopes of treatment valueless. The most promising cases are those for persons of active temperaments, with good circulation, thin skins, healthy gums, and limpid oral secretions.

### AFTER-TREATMENT.

I have now, in conclusion, to direct your attention to the after-treatment which may become necessary. It is presumed that the judicious practitioner has made careful selection of the cases to be treated conservatively, and that he will early decide upon the evident condition of those which preclude recovery. As previously indicated to you, some of the most promising cases will not yield to treatment. This consideration requires close observation of the cases for some time after treatment. The patient should be instructed to return for consultation should reflected pain occur or should the tooth become impatient of cold applications. If this should be apparent, it is a sign of needed care to avert increased disturbance.

A most marked form of reflected pain is felt in the ear, and this frequently appears before the temperature sense has become aggravated. So much importance should be attached to this symptom of pulp disturbance that the first question to be asked a patient appearing with pain, or on approaching a suspected pulp, is, Have you had any pain in the ear of that side? Reflection to the ear occurs often long in advance of similar pain in other branches of the third pair.

In any case where the tooth has been impressed by cold, either before the treatment or afterwards, an application should be made to the gum over the tooth, of tincture of aconitum root, two parts, chloroform, one part. The mode of application is important. A pledget of cotton or muslin to cover an area of one-half by three-fourths of an inch should be filled with the prescription, then squeezed out nearly to dryness between folds of a napkin to prevent an excess flowing over the mouth and with the saliva entering the fauces, to which it is extremely irritating as well as unnecessarily medicating the patient. Before the pledget is applied the surface of the gum should be cleansed of the coat of mucus covering it, otherwise the remedy will fail to come in contact with the membrane. It is equally important that dryness of the surface be secured. This application should be maintained for from twelve to fifteen seconds. If allowed to remain too long upon the part, ulceration is established. The general after-treatment consists in the repeated application of aconitum, the application not being made at the same point more frequently than after intervals of forty-eight hours. When it is desired to increase the counter-irritation, the gum may be scarified very superficially by quick, light movement of a small scalpel. The patient should be instructed to avoid subjecting the tooth to extremes of temperature in either direction. The control period of conservatively-treated cases is within the first fortnight after the capping.

I have, however, had them appear after several years of complete quiescence with initial disturbances, and then yield to treatment. It sometimes becomes necessary to open the cases and recap. This usually occurs when in reviewing the case it is considered that some oversight has occurred. There may have been two exposures. The cap may not have completely covered the exposed part. There may have become compression from forcing the cap. It may have been displaced, the case may be determined to go down the gamut of irritation, and in despair we sterilize again and make another trial.

The most careful records of cases should be kept with a relation of the condition and of the controlling symptoms. These records should be methodically preserved in a book kept for this purpose.

Should subsequent irritation occur, a new diagnosis may be formed from the recorded facts and the new conditions. I am careful to have this class of cases carried forward to the examination chart at each recurring periodic examination of the teeth. They are marked in symbol with red ink to prevent the unnecessary removal of temporary fillings, to explain the reason for their presence, and thus to avoid the accident of an unnecessary uncovering of the pulp in such cases.

